# The Stressed Heart: A Case Report of Takotsubo Cardiomyopathy in a Patient With Known Coronary Artery Disease

**DOI:** 10.7759/cureus.36277

**Published:** 2023-03-17

**Authors:** Mike Ghobrial, Abhinav Karan, Michael Omar, Gladys Velarde

**Affiliations:** 1 Internal Medicine, University of Florida College of Medicine – Jacksonville, Jacksonville, USA; 2 Cardiology, University of Florida College of Medicine – Jacksonville, Jacksonville, USA

**Keywords:** stress cardiomyopathy, apical dyskinesis, heart failure with reduced ejection fraction, cardiac chest pain, coronary artery disease, takotsubo cardiomyopathy

## Abstract

Takotsubo cardiomyopathy and acute coronary syndrome are often clinically indistinguishable, making their differentiation challenging for physicians. We present a case of a 65-year-old female who presented with acute chest pain, shortness of breath, and a recent psychosocial stressor. This is a unique case in which our patient, with known history of coronary artery disease and recent percutaneous intervention, favored a misleading initial diagnosis of non-ST elevation myocardial infarction.

## Introduction

Takotsubo cardiomyopathy (TCM) is a rare clinical entity that is characterized by systolic dysfunction, primarily affecting the left ventricle. TCM accounts for approximately 0.7-2.5% of patients presenting with troponin-positive suspected acute coronary syndrome (ACS) [1]. The pathophysiology of TCM remains unclear, and several mechanisms elucidating its cause have been proposed. The leading hypothesis suggests that TCM is caused by a surge of catecholamines in response to a physiologic stressor, ultimately resulting in myocardial stunning [1]. It has been described in the literature that elevated levels of circulating catecholamines can disrupt the cardiac microvasculature, induce coronary vasospasm, and may be directly cardiotoxic [1-3]. The diagnosis presents a challenge when also facing a patient with known coronary artery disease. Here we discuss a 65-year-old female who presented for acute onset substernal chest pain and shortness of breath. Findings on her transthoracic echocardiogram (TTE) imaging preceded notable electrocardiogram findings for several days. She was admitted for a presumed non-ST elevation myocardial infarction (NSTEMI), but ultimately was diagnosed with Takotsubo cardiomyopathy.

## Case presentation

**Figure 1:**
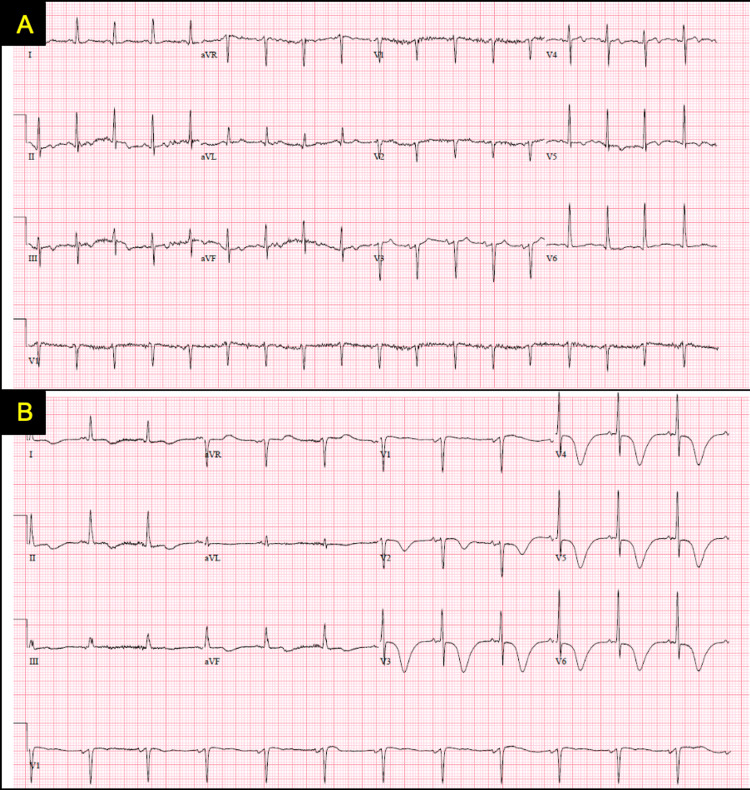
Electrocardiograms of the patient A) 12-lead electrocardiogram on admission showing sinus tachycardia, and non-specific T-wave inversions. B) A repeat 12-lead electrocardiogram showing diffuse deep T-wave inversions, and newly prolonged QTc interval to 584 ms.

A 65-year-old female with a history of coronary artery disease (CAD), heart failure with preserved ejection fraction, chronic obstructive pulmonary disease, hypertension, tobacco dependence, and prior polysubstance use disorder, presented for acute onset chest pain and shortness of breath. She was compliant with her dual antiplatelet therapy (DAPT) following percutaneous coronary intervention (PCI) to the left anterior descending (LAD) artery just two months prior to the presentation. She had a 15 pack years history of smoking tobacco. She denied use of illicit drugs. She reported a profound increase in anxiety level due to a destructive family altercation. On initial evaluation, blood pressure was 191/126 mmHg, heart rate was 87 beats per minute (bpm), oxygen saturation of 98% on room air, and temperature of 97.7 °F. Her cardiac and respiratory examinations were unremarkable. Notably, her respiratory viral panel was positive for COVID-19 infection. Laboratory testing revealed an elevated zero-hour high sensitivity troponin of 63 ng/L (reference range: <14 ng/L) with continued rise to 152 ng/L in one hour, and N-terminal brain natriuretic peptide (NT PRO BNP) of 5,676 pg/mL (reference range: 0-125 pg/mL). Her comprehensive metabolic panel was unremarkable. The initial electrocardiogram (ECG) revealed sinus tachycardia of 102, with nonspecific T-wave abnormalities, and no ST-segment elevations/depressions (Figure 1A). Chest radiography revealed engorgement of the central pulmonary vasculature and mild bilateral prominence of the pulmonary interstitium. Transthoracic echocardiogram showed newly reduced left ventricular ejection fraction (LVEF) of 30-35%, with hyperdynamic basal myocardial contractility, with mid-distal left ventricular (LV) segment akinesis, and LV apex dyskinesis (Video 1). Two months prior, the patient had known baseline left ventricular ejection fraction of 65-70%. She was initially admitted for non-ST-elevation myocardial infarction in the setting of active coronavirus disease 2019 (COVID-19) infection. A diagnosis of TCM was initially considered, but given her known CAD, COVID-19 infection, and positive troponin trend, a NSTEMI was deemed more likely. She resumed on her DAPT and started on a heparin infusion. A repeat ECG at 26 hours since admission revealed sinus rhythm with diffuse deep T wave inversions and QTc prolongation to 584 ms (Figure 1B).

**Video 1:** Transthoracic echocardiogram Transthoracic echocardiogram revealing hyperdynamic basal myocardial contractility, with mid to distal left ventricular segment akinesis, and apical dyskinesis.

Given the characteristic TTE findings, followed by the delayed dramatic ECG changes, positive trending troponins, known history of CAD, and concomitant COVID-19 infection, a cardiac magnetic resonance image (CMRI) was initially considered, but deferred due to severe claustrophobia. She was consequently scheduled for an urgent left heart catheterization (LHC), which revealed patency of previous drug-eluting stent to the LAD and no change in her known coronary artery disease from two months prior (Video 2). Her overall clinical picture was deemed more consistent with TCM, and anticoagulation therapy was subsequently discontinued. Following supportive care, she was discharged in a stable condition and had complete resolution of her apical dyskinesis at four weeks follow up (Video 3).

**Video 2:** Left heart catheterization Left heart catheterization revealing patency of the drug-eluting stent to the left anterior descending artery placed 2 months prior to her presentation.

**Video 3:** Transthoracic echocardiogram Transthoracic echocardiogram revealing resolution of left ventricular (LV) mid-distal akinesis and apical dyskinesis.

## Discussion

Takotsubo cardiomyopathy and acute coronary syndrome are often clinically indistinguishable entities. This is a unique case in which our patient with known history of CAD and recent percutaneous coronary intervention (PCI) favored a misleading initial diagnosis of NSTEMI. Takotsubo cardiomyopathy, also known as stress-induced cardiomyopathy or transient apical ballooning syndrome, is characterized by systolic dysfunction primarily affecting the left ventricle. TCM is a rare clinical entity with an estimated prevalence ranging between 0.7-2.5% of patients presenting with troponin-positive suspected ACS [1]. While the etiology of TCM is still unclear, the prevalence of the disease is rising as more clinicians are recognizing the signs [3]. TCM is most recognized among post-menopausal women over 50 years of age, following an emotional or physical stressor [2]. Our patient endorsed a psychosocial stressor that resulted in significant psychological and emotional distress, just hours before presentation. The similarity in presentation of TCM and ACS likely leads to the underdiagnosis in many cases. Thus, amongst the imaging modalities, ECGs, and laboratory studies a careful and thorough history was crucial to this case.

The question stands whether ECG has distinguishing features between ACS and TCM, as there is a paucity of data linking a single ECG pattern to TCM. Patients with TCM may present with diverse ECG findings, posing a challenge in the differentiation based on ECG. A comparative study found that the most common acute ECG changes in TCM are ST segment elevations in the anterior leads (56%), ECGs without ST segment elevations (44%), 17% were non-specific or normal, 17% revealed diffuse T-wave inversions, and 10% with healed anterior infarctions [4]. Therefore, it is important to distinguish the temporal relationship between the clinical presentation, echocardiographic findings, and ECG findings. There is a sequence of events that has been described in the literature known as the ischemic cascade. The ischemic cascade is described to begin with an imbalance of myocardial supply and demand, producing ischemia and subsequently ventricular dysfunction followed by ECG changes, which ultimately may manifest as chest pain [5]. The distinction between the ischemic cascade and the presented case lies in the timing of her chest pain presenting first, followed by delayed ECG findings. In contrast to the ischemic cascade as relates to ACS, in which the ECG changes are first, then followed by chest pain. Consequently, it is uncertain if there is clinical significance in this temporal distinction and further research is warranted.

Echocardiography was another crucial component in the early workup of this case as there was discordance between her known coronary disease and the new wall motion abnormalities seen on TTE. Her echocardiogram displayed findings of the apical form of TCM with hyperdynamic basal myocardial contractility, mid-distal left ventricular (LV) segment akinesis and LV apex dyskinesis. Among the variants of TCM, the apical type is the most common variant comprising 81.7%, midventricular type 15%, basal type 2%, and focal type 1.5% [1]. Advanced imaging modalities such as cardiovascular magnetic resonance imaging (CMRI) have shown promise in aiding the diagnosis of TCM, while excluding other entities such as myocarditis or myocardial infarction (MI) [6]. Inflammation of the myocardium has been demonstrated to occur in the acute phase of TCM and is seen on CMRI as edema [6,7]. The area of myocardial edema seen typically corresponds and matches the area of wall motion abnormality [7]. However, CMRI cannot differentiate myocardial edema in TCM from myocardial edema due to myocarditis or MI. Therefore, utility of a gadolinium-based contrast and the technique known as late gadolinium enhancement (LGE), can aid in the differentiation of TCM and other entities. While LGE is almost always identified in MI or myocarditis, the absence of LGE is suggestive of TCM demonstrating the value of CMRI in these cases [6,7]. As such the use of CMRI was initially employed, unfortunately, due to her claustrophobia, the study was deferred.

Our patient discussed in this case presented as a “high risk” non-ST elevation myocardial infarct profile. Her initial ECG showed sinus tachycardia and non-specific T-wave inversions. With her known history of CAD, recent PCI, and positive troponins this initially deterred a clinical suspicion for TCM. As such invasive cardiac testing with LHC was necessary to diagnose TCM definitively. The International Takotsubo registry estimates 15% of those with TCM have concurrent coronary artery disease, and therefore should not impede a diagnosis of TCM [8]. Her LHC revealed patency of the stent to her LAD, and no change in her known CAD from two months prior. The overall clinical picture was most consistent with TCM, and anticoagulation therapy was subsequently discontinued.

## Conclusions

Overall, TCM remains a challenging diagnosis that is infrequently considered in the setting of known CAD. This case highlights the importance of a holistic approach to the patient that presents with chest pain, shortness of breath, and a newly decreased LVEF. Therefore, physicians should have a high clinical suspicion for TCM in the appropriate setting when presented with a postmenopausal female and recent psychosocial stressors. Further research is needed to elucidate the etiology, reason for variance in the clinical presentations, and pathophysiology of Takotsubo cardiomyopathy.
